# The Effect of the Morphology of Coarse Aggregate on the Properties of Self-Compacting High-Performance Fibre-Reinforced Concrete

**DOI:** 10.3390/ma11081372

**Published:** 2018-08-07

**Authors:** Krzysztof Ostrowski, Łukasz Sadowski, Damian Stefaniuk, Daniel Wałach, Tomasz Gawenda, Konrad Oleksik, Ireneusz Usydus

**Affiliations:** 1 Faculty of Mining and Geoengineering, AGH University of Science and Technology, Al. Mickiewicza 30, 30-059 Cracow, Poland, walach@agh.edu.pl (D.W.); gawenda@agh.edu.pl (T.G.); koleksik@agh.edu.pl (K.O.); 2 Faculty of Civil Engineering, Wrocław University of Science and Technology, Wybrzeże Wyspiańskiego 27, 50-370 Wrocław, Poland, lukasz.sadowski@pwr.edu.pl (Ł.S.); damian.stefaniuk@pwr.edu.pl (D.S.); 3 The State School of Higher Education in Chełm, ul. Pocztowa 54, 22-100 Chelm, Poland, iusydus@pwsz.chelm.pl

**Keywords:** self-compacting concrete, morphology, coarse aggregate, flowability, fibre-reinforcement

## Abstract

When understanding the effect of the morphology of coarse aggregate on the properties of a fresh concrete mixture, the strength and deformability of self-compacting high-performance fibre-reinforced concrete (SCHPFRC) can be seen to be critical for its performance. In this research, regular and irregular grains were separated from granite coarse aggregate. The morphology of these grains was described while using digital image analysis. As a result, the aspect ratio, roundness and area ratio were determined in order to better understand this phenomenon. Then, the principal rheological, physical, and mechanical properties of SCHPFRC were determined. The obtained results indicated that the morphology of the grains of coarse aggregate has an impact on the strength and stiffness properties of SCHPFRC. Moreover, significant differences in the transverse strain of concretes were observed. The morphology of the coarse aggregate also has an impact on the rheological parameters of a fresh concrete mixture. To better understand this phenomenon, the hypothesized mechanism of the formation of SCHPFRC caused by different morphology of coarse aggregate was proposed at the end of the article.

## 1. Introduction

An increase of interest in the use of self-compacting concrete (SCC) in civil engineering has recently been observed. It is still evolving and it covers an ever-wider range of applications and properties. SCC spreads smoothly around reinforcement due to its flowability and the small size of aggregate. Kostrzanowska-Siedlarz and Gołaszewski highlighted that the composition of the high performance SCC effect its rheological properties [1]. However, the methods of mixing and curing, together with the resistance of SCC to environmental conditions, are still under development [2]. Recently, the application of steel fibers has enabled the utilization of self-compacting high performance fibre-reinforced concrete (SCHPFRC). The use of steel fiber is essential to secure high strength and ductility in the production of ultra-high performance concrete [3]. As pointed out by Wu et al. [4] the type of reinforcement and bond properties determines the efficiency of use of steel fibres.

In the concrete industry, most aggregates used in SCHPFRC mix designs are built of magmatic rocks. The morphology of aggregate is closely associated with the types of rocks, its mineralogical composition, and the allied crushing mechanism. Rajan and Singh [5] shown that the type of crusher plays an important role in the manufacturing of aggregates and the obtaining of different morphology parameters. The most desired, due to their lower specific surface area, are regular particles with a spherical morphology. Furthermore, flat and elongated particles have a higher specific area and a tendency to orientate in one plane. The high influence of irregular particles on the durability of aggregates occurs when their content varies from 25 to 50% in the total aggregate content. As pointed out by Zieliński [6], the durability of basalt aggregate is getting lower by 55% when the share of irregular particles is equal to 50% in the total aggregate content. Therefore, both the granulometric distribution and the morphology of particles may have an effect on the quality of SCHPFRC.

Existing studies have demonstrated that coarse aggregates of the same content, type and surface texture, but with different aspect ratios and angularity indices, have an impact on the mechanical behavior of hardened concrete [7,8]. Preliminary research of the influence of coarse aggregate morphology on the properties of the self-compacting high-performance concrete was presented by Ostrowski [9]. However, in this study, three unreinforced concrete mixtures were analyzed. Moreover, aggregate surface characteristics become more pronounced with an increased matrix quality [10,11,12]. Qian et al. [13] presented a geometrical model that allows the composite structure of mortar and concrete with real-shape aggregates to be simulated. Han et al. [14] proposed 2D cross-section image analysis to evaluate the characteristics of coarse aggregate and its distribution in the concrete mix. Cepuritis et al. [15] analyzed the three-dimensional (3D) morphology of concrete fine aggregates while using the vertical shaft impact (VSI) crushing system. They proposed the micro-Flakiness Index (mu FI) to characterize the morphology of the fine crushed concrete aggregate particles. Effects of coarse-aggregate morphology on the shear performance of aggregate-based materials was documented by Zhang et al. [16]. The quality and characteristics of coarse aggregate have a significant influence on the estimation of the strength of concrete [17,18]. According to de Brito et al. [19], shape regularity of the aggregate is one of the major factors that affect the influence of aggregates on concrete quality. Özturan and Cecen [20] analyzed the effect of coarse aggregate type on mechanical properties of concretes with different strengths. The results have shown that the strength, stiffness, and fracture energy of concrete depend on the type of aggregate, especially for high-strength concrete. Brandt [21] found that the compressive strength of concrete is different when made with rolled or crushed natural aggregates. The role of coarse aggregate in the design and behavior of SCC was noted in the literature [22]. In comparison to normal concrete, the mix design of highly flowable concrete is more complex and it should ensure that the mixture could develop adequate static and dynamic stability. The selection of aggregate is a primary factor for the mix design and mixture optimization of flowable concrete.

Apart from the cement matrix, the coarse aggregate that is used in a mix seems to be crucial for the behavior of SCHPFRC. Most of the researchers working on SCHPFRC concentrate on the optimization of the cement matrix, the used fibers, and the minimizing of the water/binder content. Even though there is still a good method of optimizing the mix, the chances of such attempts are getting smaller. More advanced optimization methods at micro- and nano-levels should be employed. In the authors opinion, there is usually a lack of information regarding the morphology of used aggregate. In most research, only granular analysis and the origin of aggregate is presented. The chemical composition is also sometimes analyzed. This does not make the experiment replicable by other researchers. In most cases it is enough to change the origin of the aggregate to obtain completely different results. In the authors’ opinion, other factors should be taken into account. Does the morphology of the aggregate matter? What about its regularity? Knowledge about these factors is crucial to understand the behavior of SCHPFRC. This knowledge will allow for the properties of SCHPFRC to be optimized in the future. When considering the above, the goal of this research will be to answer the question of whether the morphology of coarse aggregate has an impact on the behavior of self-compacting high-performance fibre-reinforced concrete.

## 2. Materials and Methods

### 2.1. Preparation of Coarse Aggregate

In order to prepare the coarse aggregate, the sieving system with a multi-product screen and slotted sieve, with the possibility to return the subscreen product to additional comminution process, was applied [23]. The aggregate production system has been patented [24].

The designed circuit makes it possible to obtain final aggregates with a content of irregular particles of no more than 2–3%. Even with the use of only one jaw crusher, such a system requires the application of vibrative screens with sieves of quadratic mesh and slotted mesh, which cooperate with the crusher that is located in the first or second stage. The task of a multideck screen is to classify aggregates into the narrow particle fractions that occur in a single deck multi-product screen with a slotted sieve. The irregular particles are then sieved (lower product) and returned again to comminution. The irregular particles were comminuted in the same crusher or on the secondary stage of crushing. The contents of irregular particles depend on the capacity of the screen with the slotted sieve, and also on the relation between the narrow particle fraction range and the size of the slot in the sieve. This sieve was selected to be about half of the maximum size of a certain fraction’s particle (d_max_/2). Because the share of irregular particles decreases with an increase in particle fractions, and the efficiency of screening grows for coarser particles, the sieving of irregular particles in coarser fractions will then be easier and more efficient. This is why the finest particle size fractions (6.3–8 mm) were investigated in order to determine the maximum contents of the irregular particles that could occur in the prepared aggregate.

The material comminuted in a jaw crusher of L44.41 type was classified on a vibrative two-deck screen in a way that ensures the selection of narrow particle fractions of 6.3–8 mm. This fraction contained about 76% of regular particles and 24% of irregular ones. As a result of sieving the 6.3–8 mm fraction, the contents of the regular particles (the so-called cleanness of beneficial particles) was raised from 76.1 to 97.2%, which means that this fraction will only contain about 2.8% of irregular particles in the final product instead of 24% with the technological screening efficiency, according to Hancock equal to 92.5%.

**Figure 1:**
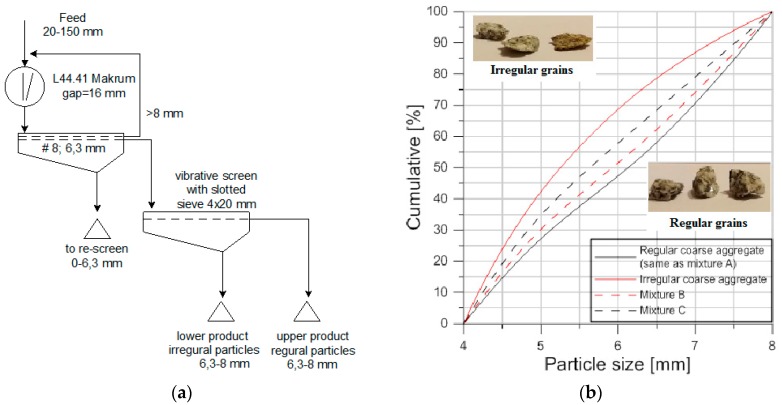
(**a**) Scheme of granite comminution in a L44.41 type jaw crusher that divides aggregates into regular and irregular particles (size fraction 6.3–8 mm) and (**b**) also Particle size distribution of the prepared coarse aggregate.

In order to prepare the coarse aggregate technological circuit for the production of granite aggregates, with regular and irregular particles in narrow size fractions of 4–5 mm, 5–6.3 mm and 6.3–8 mm, was built (Figure 1a).

### 2.2. Experimental Setup

All of the concrete mixtures were made with the same proportions of ingredients and a constant water/binder ratio (see Table 1). Three compositions of concrete were studied (A, B, and C). Composition A contains only regular aggregates (950 kg/m^3^), composition B contains 760 kg/m^3^ of regular and 190 kg/m^3^ of irregular aggregates, and composition C contains 475 kg/m^3^ of regular and 475 kg/m^3^ of irregular aggregates. In all of the compositions (A, B, and C), a total content of 950 kg/m^3^ of coarse aggregate was used. The detailed mix designs for all of the specimens are given in Table 1. All of the mixes were prepared using Portland Cement type I 52.5R (Górażdże, Poland), a 0.28 water/binder ratio, SikaFume additive in fine-powder form based on silica fume technology, and superplasticiser Sika ViscoCrete-20 HE (Sika Poland sp. z.o.o., Warsaw, Poland) based on an aqueous solution of modified polycarboxylates. Separated granite coarse aggregate (Kamienna Góra, Poland) and fine aggregate (Brzegi, Poland) were used. The dimensions of steel fibres depend on the fraction of coarse aggregate. According to Katzer [25], the maximum length of fibres should be less than double value of maximum fraction of coarse aggregate in concrete mixture. In addition, due to specimen’s dimension, authors wanted to minimalize the effect of fibres in concrete. Therefore, the steel reinforcement made of 14 mm length simple steel fibres with a 0.3 mm outer diameter (*R*_m_ > 1020 MPa) was chosen.

The only variable was the morphology of the coarse aggregate. The feed material, which was subjected to a process of shredding in a crusher, was granite (fraction *ϕ* from 20 mm to 150 mm). The resulting product was properly separated by using slotted sieves in order to receive regular and irregular grains according to EN 933-4 [26]. The following narrow size fractions were extracted: 4–5 mm, 5–6.3 mm, and 6.3–8 mm. These fractions were separated by using sieves with spacing between bars of 2.5 mm, 3.15 mm, and 4 mm, respectively. The upper products on the slotted sieve are regular grains, while the lower products are irregular grains. The study only used grain sizes of *ϕ* 4–8 mm. This range of grain size allows for obtaining self-compacting high-performance fibre-reinforced concrete (SCHPFRC). The bulk density of coarse aggregate is *ρ*_a_ = 2.64 g/cm^3^. Figure 1b presents the particle size distribution curve of:

- regular coarse aggregate (same as mixture A),
- irregular coarse aggregate,
- mixture B (80% of regular and 20% of irregular coarse aggregate, this ratio is most commonly found in concrete aggregates and results from the widespread crushing systems), and
- mixture C (50% of regular and 50% of irregular coarse aggregate).

Regular and irregular coarse aggregate is different in the content of each narrow size fractions (Figure 1b). The largest part of the regular coarse aggregate is size fraction 6.3–8 mm (46.4%) and the smallest part is fraction 5–6.3 mm (26.4%) and 4–5 mm (27.4%). While for irregular coarse aggregate the largest part is fraction 4–5 mm (42.4%) and the smallest part is fraction 6.3–8 mm (25.1%). The content of each narrow fractions in regular and irregular coarse aggregate is determining the particle size distribution for mixture B and C.

A total of 36 cylindrical specimens were manufactured in laboratory conditions and were tested under uniaxial compression. The compression tests have been performed according to EN 12390 [27]. All of the specimens were 118 mm in height and had a diameter of 59 mm. With each type of concrete mixture, twelve specimens were made; four of them were tested after 3, 7, and 28 days. Within the first 28 days, the concrete specimens were immersed in water with a temperature of 20 °C.

**Figure 2:**
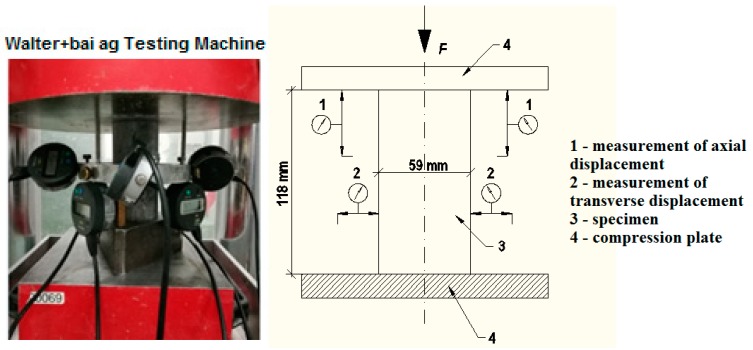
Layout of extensometers on the specimens.

The early age properties of SCHPFRC were tested by using a slump flow test, according to EN 12350 [28]. The viscosity of the fresh mixture was determined when the flowing concrete mixture reached a diameter of 500 mm. The density of the concrete was calculated by dividing the mass by the volume of the specimens. The compression tests were conducted using a 3000-kN capacity high-stiffness testing machine. The modulus of elasticity *E* was determined according to EN 12390 [29]. The density, porosity and absorptivity of concrete was established from weight loss observations. First, after 28 days three specimens from each type of concrete mix were weighed. Then these specimens were immersed and kept in water for 7 days to fill the pores. After that the specimens were weighed. All the specimens were then dried at the temperature of 105 °C for 7 days and weighed again. The research was carried out at an air temperature of 20 ± 2 °C and humidity of 60 ± 2%, with the constant axial strain rate of the samples in all of the experiments being approximately 3 × 10^−5^ [s^−1^]. The measurement of the axial force was carried out by the means of a force transducer, while the displacements were measured by an extensometer. Radial and axial displacements were determined using the measurement of all the specimens’ dimension changes, where the extensometer was mounted directly between compression plates. The layout of the extensometer on the specimens is shown in Figure 2.

### 2.3. Microscale Laboratory Test

One of each concrete sample (A, B, and C) after 56 days of curing was tested using X-ray micro-computed tomography (micro-CT). A scanner with a charge-coupled device (CCD) camera resolution of 11 Mp (GE phoenix v|tome|x s, General Electric Measurements, Boston, MA, USA) with a GE DXR250RT detector was used for this purpose. To scan the samples, the following parameters were adopted: 240 kV source energy, a 0.5 mm Cu filter, an angle rotation of up to 360° at 0.35° step, 333 ms exposure time, and a resolution of 60 μm/px (micrometer per pixel). The reconstructions of the obtained projections were made using Phoenix Datos|x CT software (General Electric Measurements, Boston, MA, USA). Image processing and analyses were performed using CTAnalyser software version 1.16 (SkyScan, Kontich, Belgium). 

Since the aggregate has a very similar attenuation coefficient as the cement matrix, it is hard to distinguish (segment) it on micro-CT scans. However, the aggregate morphology is analyzed thoroughly in Section 5 while using a different method. Therefore, micro-CT was used to investigate the influence of the coarse aggregate type on the structure of air voids and distribution of steel fibres. The analysis was preceded by filtering and by binarization of the images (for more details see e.g., [30]). Because of the good contrast in the attenuation coefficient values, air voids, as well as steel fibres, were easily segmented from a solid phase with a simple thresholding.

After segmentation, 3D micro-CT images were used to analyze the spatial distribution, size, and shape of the air voids. The size of the air voids was analyzed using a volume-equivalent sphere diameter measure, which was evaluated by replacing the volume of a given air void (*V*_p_) by the equivalent perfect sphere of diameter *D*_eq_, and can be defined as:

(1) $$D_{\mathrm{eq}}=\sqrt[{3}]{\frac{6\cdot V_{p}}{\pi }}.$$

In turn, the shape of the air voids was characterized using the sphericity (*ψ*) measure, which can be defined as the ratio of the surface area of a sphere (with the same volume as the given air void) to the surface area of the air void (*A*_p_):

(2) ψ=π3⋅(6⋅Vp)23Ap.

**Figure 3:**
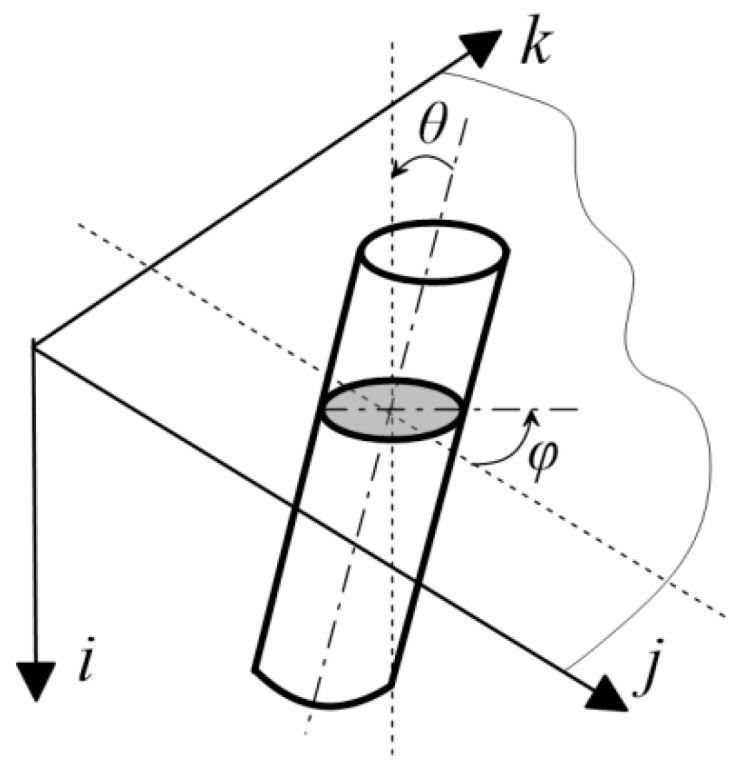
In-plane (*ϕ*) and out-of-plane (*θ*) orientation angles of an inclined steel fibre.

In order to investigate the influence of the coarse aggregate type on the distribution of steel fibres, their spatial orientation was evaluated. In particular, the in-plane (*ϕ*) and out-of-plane (*θ*) orientation angles of steel fibres (see Figure 3 for notation; notation is adopted from Nunes et al. [31]) were determined.

## 3. Morphological Characterization of Coarse Aggregate

**Figure 4:**
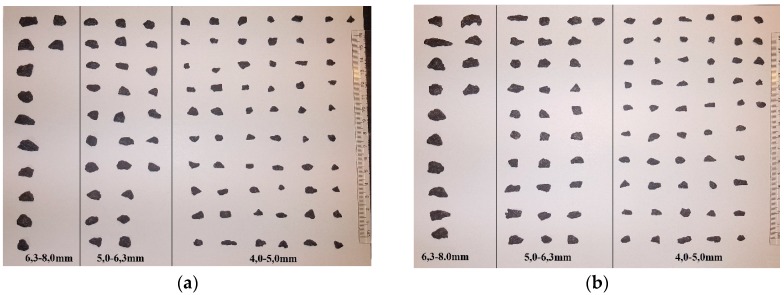
Top view of: (**a**) regular coarse particles and (**b**) irregular coarse particles.

The granite consists of many minerals that differ in color. Consequently, to facilitate the digital image analysis, the particles’ aggregate surface was painted using black spray. Representative samples of 100 regular and 100 irregular particles of coarse aggregate were selected for the analysis while maintaining the percentage mass fraction of particular particle size classes. The particles were laid out on a white background, according to their particle size class, and high quality photos were taken with adequate lighting to minimize shadows (Figure 4).

**Figure 5:**
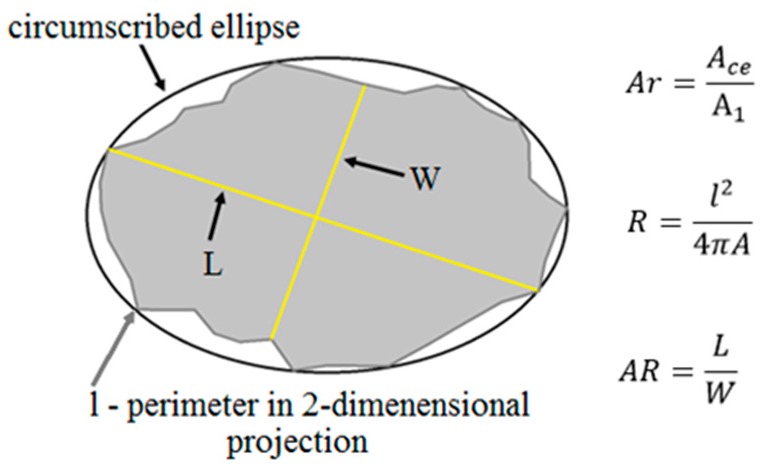
Circumscribing ellipse of an aggregate image.

Open source digital image analysis software ImageJ v.1.51w. (National Institutes of Health, Bethesda, MD, USA) was used to measure the particles’ dimensions and to determine selected morphological factors, according to Xianglin Gu et al. [7], as presented also in Figure 5:

- Area ratio (*A*_r_) describes the form of particles in a 2-dimensional system. It is defined as the ratio of the area of a circumscribing ellipse (*A*_ce_) to the area of a particle (*A*_1_). The value of the area ratio for a circle or elliptical image of a particle is equal to 1.
- Roundness (*R*) describes how closely the morphology of the particle approaches the circle. The value of roundness is equal to or greater than 1.
- The aspect ratio (*AR*) of a particle is used to describe the form of particles in a two-dimensional system. It is defined as the ratio of the particle’s length (*L*) to its width (*W*). The aspect ratio of a circle and equilateral polygon is equal to 1.

**Figure 6:**
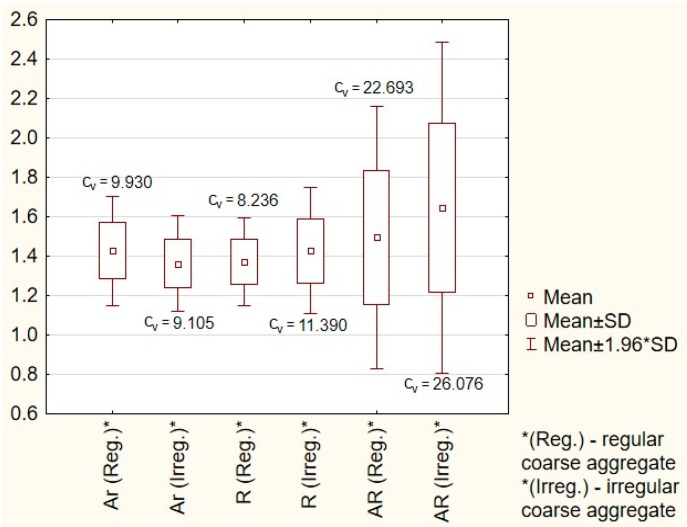
Box and whisker plot of selected morphological factors.

For regular and irregular coarse aggregate, the following statistical parameters were determined: mean values, standard deviations, and coefficient of variations of the selected morphological factors. In the next stage of the investigation, the types of distributions of the analyzed morphological factors were analyzed. The statistical parameters are presented in Figure 6. 

**Figure 7:**
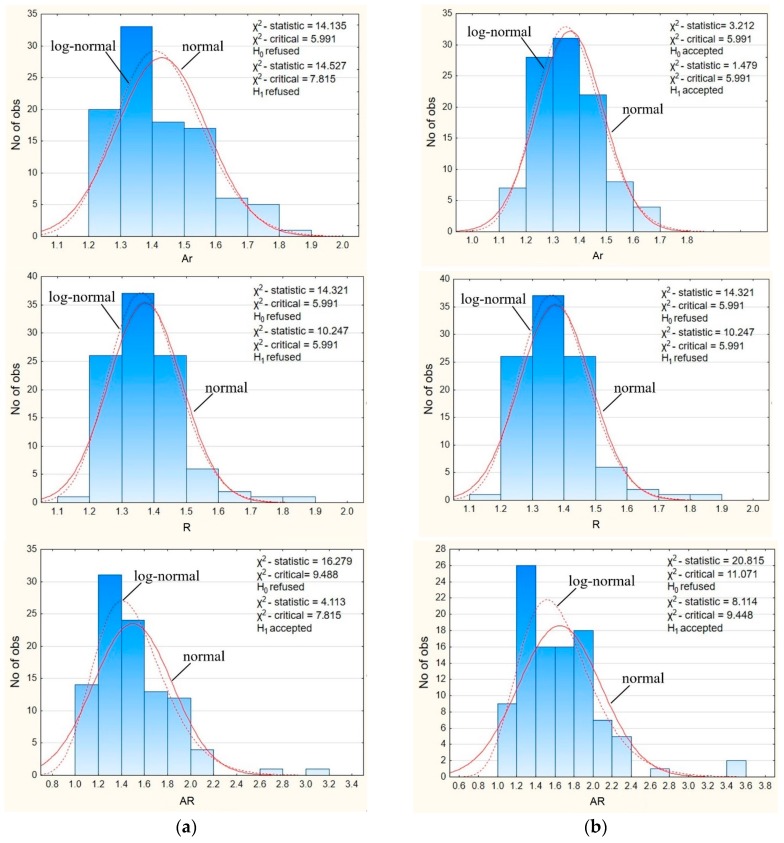
Histograms and Chi-square for the normal and log-normal distributions of area ratio (*A*_r_), roundness (*R*), and aspect ratio (*AR*) of: (**a**) regular coarse particles and (**b**) irregular coarse particles.

The value of each analyzed morphological factor for a circle is equal to 1. When analyzing the results, it can be noticed that for regular coarse aggregate the values of aspect ratio and roundness are closer to 1 than for the irregular coarse aggregate, which indicates that the morphology of regular coarse aggregate is closer to the shape of a circle than the shape of irregular coarse aggregate. Since the value of the area ratio for a circle or an elliptical image of a particle is 1, it was assumed that the lower mean value of the area ratio for irregular coarse aggregate when compared to regular coarse aggregate is caused by the specificity of this morphological factor. Therefore, the area ratio cannot be used to study the influence of the form of aggregate on the mechanical properties of concrete. The coefficient of variation (*c*_v_) of each morphological factor ranges from 8.236 to 22.693% for regular coarse aggregate and from 9.205 to 26.076% for irregular coarse aggregate, which indicates that the regularity of the coarse aggregate affects the morphological factors. The normal or log-normal distributions of a selected morphological factor were verified with the usage of Pearson’s Chi-squared test [32]. Two hypotheses were assumed: H_0_ when the distribution is a normal distribution and H_1_ when the distribution is a log-normal distribution. Results are presented in Figure 7.

It is visible from Figure 7 that the assumption of the normal distribution was only fulfilled for the area ratio index of irregular coarse aggregate, whereas the assumption of the log-normal distribution was fulfilled for the aspect ratio of regular coarse aggregate and for the aspect ratio and area ratio of irregular coarse aggregate. It is assumed that the obtained results are caused by the separation of the aggregate, in particular, particle size classes into regular and irregular coarse aggregate. It proves that regular grains of coarse aggregate have different parameters than irregular grains. In particular, regular grains have a more circular shape, as well as a higher area ratio and aspect ratio than irregular grains.

## 4. Results and Discussion

### 4.1. Properties of Fresh Concrete Mixture

**Figure 8:**
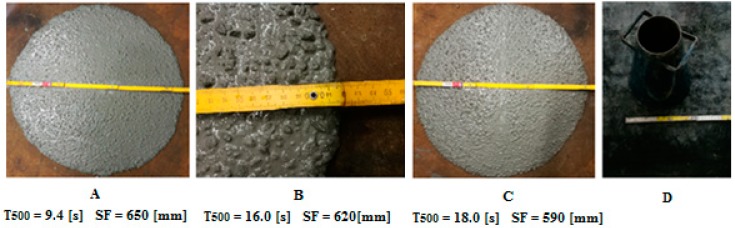
Slump Flow (SF) test for concrete mixtures (**A**–**C**); and Instrumentation to Slump Flow test (**D**).

The rheological parameters of fresh concrete mixtures are presented in Figure 8. It can be observed that the concrete mixture with only regular grains has a higher slump flow than mixture B and C. It is associated with the content of irregular grains—these have a different friction angle than the regular grains. The plastic viscosity is higher in the case when both regular and irregular grains are used in concrete mixtures. In each concrete mixture, there was no leakage of cement paste—this proves that all of the concrete mixtures were made properly. Moreover, sorting of the components did not occur.

### 4.2. Selected Physical and Mechanical Properties of Hardened Concrete

**Figure 9:**
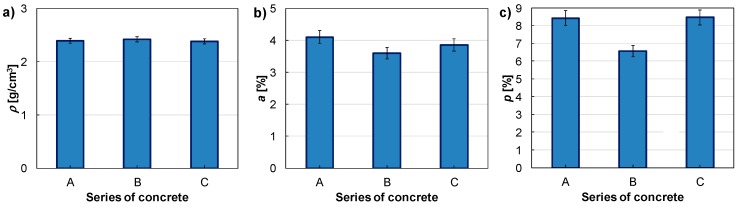
Selected physical properties of tested concretes: (**a**) density; (**b**) absorptivity; and (**c**) total porosity.

In Figure 9, the selected physical properties of the tested concretes are highlighted.

The water absorption and porosity were extracted from the same samples. Concrete density is similar in each type of concrete. Concrete absorptivity is the highest in concrete A, and the lowest in concrete B. Total porosity is similar in concrete with regular grains and in concrete with a 50% content of irregular grains, and it is higher than 8%. The lowest total porosity was obtained in the concrete with 20% of irregular grains. Between water absorption and porosity any relationship has not been observed.

**Figure 10:**
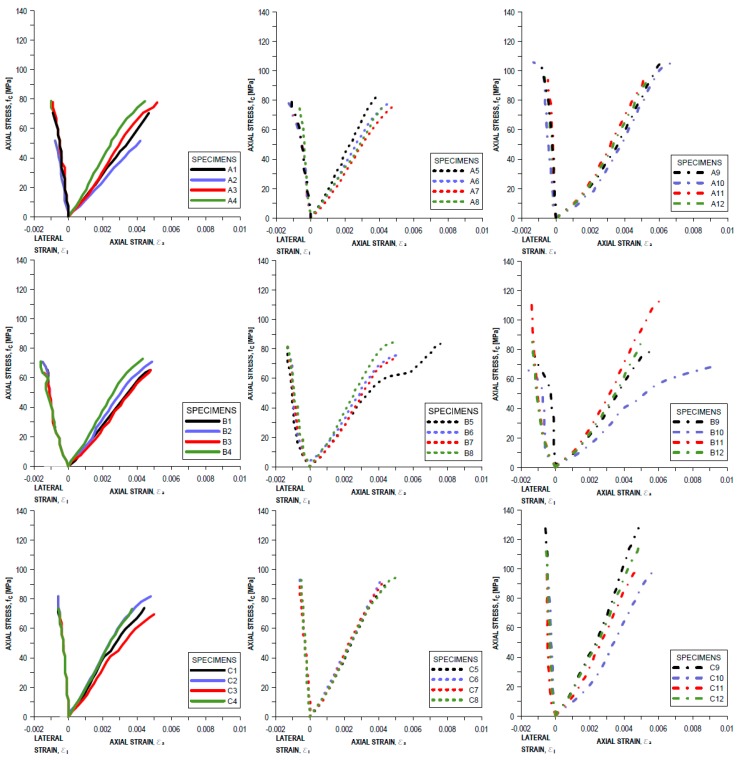
Axial and lateral stress–strain relationship modes for tested concretes: A, B, and C.

**Figure 11:**
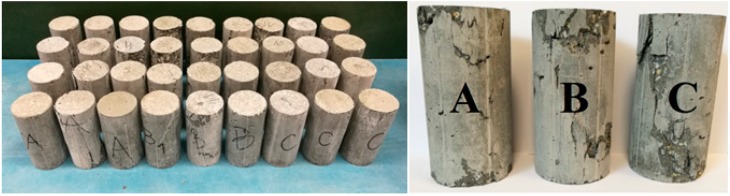
All of the samples after compression tests and the typical failure modes for tested concretes A, B, and C.

There were differences in the compressive strength and Young’s modulus of the concrete for different morphology of grains (Table 2). The average compressive strength at 28 days in concrete C is higher by 10% than concrete A (with regular grains only), and 27% higher in comparison to concrete mixture B. The Young modulus was designated without preloading cycles. Therefore, the results are qualitative. The highest value of average Young modulus has been performed at 28 days in the case of concrete ‘C’ and was 24.09 GPa. During the compression tests, vertical cracks were observed on the surface of the tested SCHPFRC elements. It is similar to the crack pattern that was observed in ordinary concrete cylinders. The mechanical characteristics, axial and transverse strains-axial stress for all the specimens are presented in Figure 10. Representative typical failure modes for the tested concretes at 28 days are presented in Figure 11.

The axial and transverse strains are different depending on the morphology of the aggregate. The average axial strain during fracture was 5.62‰, 4.96‰, and 4.72‰ in the case of concrete B, A, and C, respectively. The average transverse strain was the smallest in concrete C—0.58‰, and higher in concrete A and B; where it was 0.96‰ and 1.4‰, respectively. The presence of a small amount of irregular coarse aggregate (concrete B) could cause interference in the structure of the concrete mixture. These grains are arranged perpendicular to the direction of concrete laying, and therefore have a lower mechanical strength. Additionally, air bubbles can accumulate under their surface, which can further reduce the strength and lead to the higher values of axial and transverse strains. Due to the control of compression tests with the use of the constant axial strain rate, the sudden failure of specimens did not occur.

### 4.3. Microscale Investigation

**Figure 12:**
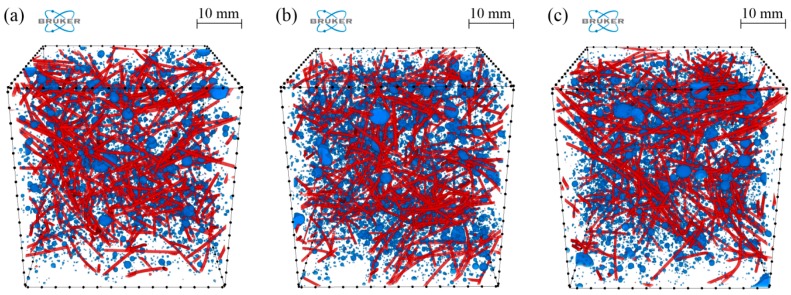
Segmented air voids (blue) and steel fibres (red) for samples: (**a**) A; (**b**) B and (**c**) C.

**Figure 13:**
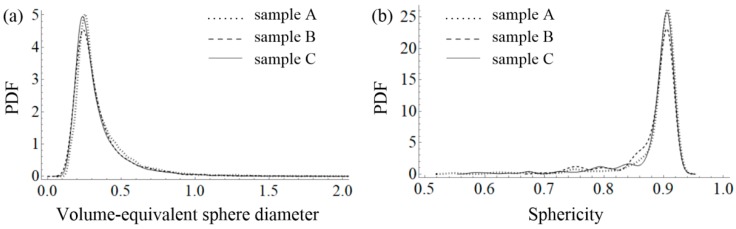
Probability density functions of: (**a**) volume-equivalent sphere diameter and (**b**) sphericity for samples A–C.

In order to avoid the boundary effect, only the middle part of each sample, called the volume of interest (VOI), was analyzed, i.e., a cube with a side of 36 mm. Segmented air voids, as well as steel fibres for samples A–C, are presented in Figure 12 (CTVox software version 3.2.0 (SkyScan, Kontich, Belgium) was used for visualization purposes). The distribution of the size of air voids is presented in Figure 13a as a probability density function (PDF) of the volume-equivalent sphere diameters of the air voids. In turn, the shape of the air voids is presented in Figure 13b as the PDF of sphericity. It can be seen that no significant differences can be noticed for all of the investigated samples.

**Figure 14:**
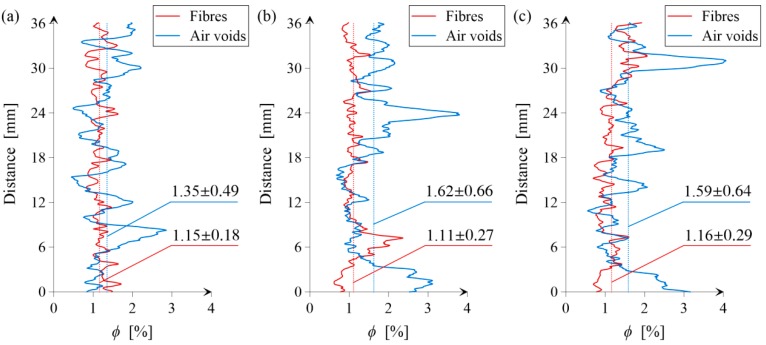
Air void and fibre contents along the height of sample: (**a**) A; (**b**) B and (**c**) C. The charts are supplemented with the mean values and standard deviations (*µ* ± *σ*).

In the next step, the distribution of air voids and steel fibres was analyzed, i.e., the volume fraction (*ϕ*) of air voids and steel fibres as a function of sample height (distance from the bottom part of the investigated VOI). The results are set together in Figure 14. It can be seen that, in the case of the volume fraction of air voids, the results for samples B and C almost coincide. In turn, the average volume fraction of the air voids for sample A is lower and it is equal to 1.35% in comparison to samples B and C, i.e., 1.62% and 1.59%, respectively. The volume fraction of fibres is almost the same for all the samples (1.15%, 1.11%, and 1.16% for samples A, B, and C, respectively). However, the standard deviation (*σ*) for sample A is much lower, i.e., 0.18% in comparison to samples B and C (0.27% and 0.29%). The same tendency (lower *σ* value) can be noticed, but less clearly, in the case of the volume fraction of air voids. Therefore, the spatial distribution of both the air voids and steel fibres is more homogeneous for sample A, where only regular grains in the concrete mixtures were used.

**Figure 15:**
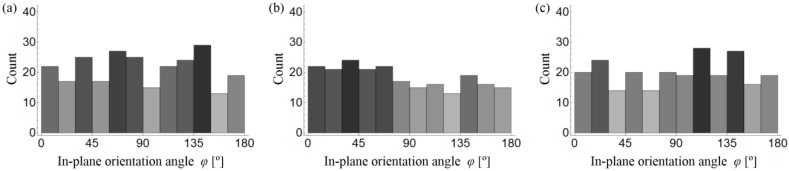
Histograms of in-plane (*ϕ*) fibre orientation for sample: (**a**) A; (**b**) B and (**c**) C.

**Figure 16:**
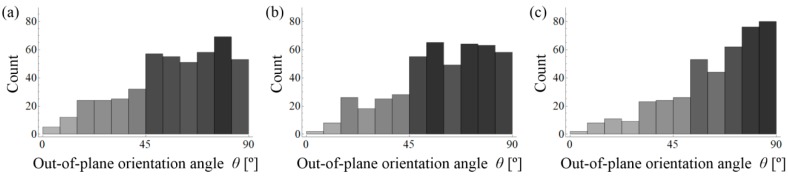
Histograms of out-of-plane (*θ*) fibre orientation for sample: (**a**) A; (**b**) B and (**c**) C.

Histograms of in-plane (*φ*) fibre orientation for samples A–C are presented in Figure 15. When comparing the results between all of the samples, no significant differences can be seen. Moreover, the results indicate no privileged in-plane orientation of fibres. The histograms of out-of-plane (*θ*) fibre orientation for samples A–C are presented in Figure 16.

It is clearly visible from Figure 15 and Figure 16 that the fibres are mainly oriented horizontally (*θ* → 90°) rather than vertically (*θ* → 0°) for all of the samples. This is caused by the gravity force and the fact that the density of steel fibres is much higher than the density of the cement matrix and aggregate. However, what is most remarkable is that the horizontal orientation is most visible for sample C, in which the highest number of fibres are oriented between 75° and 90° in comparison to samples A and B, where this interval is wider and roughly between 45° and 90°. Therefore, a small volume of irregular aggregate grains (sample B and C) results in a reduction of the number of fibres that are oriented horizontally, and as a consequence, a reduction of the anisotropy effect caused by gravity.

## 5. Conclusions

This work presents an experimental investigation of the influence of the morphology of coarse aggregate on the properties of self-compacting high-performance fibre-reinforced concrete. The following conclusions may be drawn from the work presented in this paper:

- It has been proven that the morphology of the grains of coarse aggregate has an enormous effect on the compressive strength and stiffness of the tested concretes. This may be due to the different friction angle and better filling of the grain skeleton by the mix of aggregate containing regular and irregular grains when compared to the regular grain aggregate,
- The sharper edges of the irregular aggregates may induce more pronounced stress concentrations. In this case, it would actually lead to an improved compressive strength. The lowest transverse strains occur in the case of concrete C, which contains the highest amount of irregular aggregate,
- The morphology of the grain aggregate also has an effect on the rheological early age properties of fresh concrete mixes. Based on the presented research, it can be concluded that the appropriate selection of the morphology of the grains of coarse aggregate can affect the rheological parameters of concrete. The research will be continued in order to reduce the proportion of water and cement in relation to the share of aggregates of various morphologies,
- Digital analysis of coarse aggregate showed that the values of the aspect ratio and roundness are closer to 1 for the regular coarse aggregate than for irregular coarse aggregate, which indicates that the morphology of regular coarse aggregate is closer to the shape of a circle than to the morphology of irregular coarse aggregate,
- The morphology of the grain aggregate also has an effect on the air voids and steel fibre distribution along the height of sample. It was observed that this distribution is more homogeneous for the sample with only regular grains in the concrete mixture,
- The morphology of the grain aggregate also has an effect on the orientation of steel fibres. It was observed that a small volume of irregular aggregate grains results in a reduction of the number of fibres oriented horizontally, and as a consequence, a reduction of the anisotropy effect that is caused by gravity.
- In the future work, the effect of coarse aggregate morphology on the behavior of normal concrete and for self-compacting high-performance concrete without steel fibres will be performed. It will allow for determining the essence of this parameter in the design of this type of concretes.

## Figures and Tables

**Table 1:** Proportions of concrete mixtures.

Mix Type	Cement [kg/m^3^]	Fine Aggregate [kg/m^3^]	Regular Coarse Aggregate [kg/m^3^]	Irregular Coarse Aggregate [kg/m^3^]	Water [kg/m^3^]	Sika Fume [kg/m^3^]	Super-Plasti-Ciser [kg/m^3^]	Steel Fibres [kg/m^3^]
A	550	850	950	-	173	66	19.25	78
B	550	850	760	190	173	66	19.25	78
C	550	850	475	475	173	66	19.25	78

**Table 2:** Selected mechanical properties of the tested concretes.

Mix Type	Average Compressive Strength at 3 Days ${\bar{R}}_{c3}$ [Mpa]	Average Compressive Strength at 7 Days ${\bar{R}}_{c7}$ [Mpa]	Average Compressive Strength at 28 Days ${\bar{R}}_{c28}$ [Mpa]	Average Young Modulus at 3 Days ${\bar{E}}_{3}$ [Gpa]	Average Young Modulus at 7 Days ${\bar{E}}_{7}$ [Gpa]	Average Young Modulus at 28 Days ${\bar{E}}_{28}$ [Gpa]
A	69.90	78.73	100.91	16.55	19.31	18.82
B	68.47	79.24	87.28	15.05	15.85	15.50
C	74.75	93.43	110.94	20.03	23.53	24.09

## Nomenclature & Symbols

ρ	density, [kg/m^3^]
ρ_a_	bulk density of coarse aggregate, [kg/m^3^]
*d*_max_	maximum size of a certain fraction’s particle, [mm]
*a*	absorptivity, [%]
*p*	total porosity, [%]
*R_m_*	tensile strength of steel fibres, [MPa]
*AR*	aspect ratio, [-]
*L*	length of particle, [mm]
*W*	width of particle, [mm]
*R*	roundness, [-]
*l*	perimeter in a 2-dimensional projection, [mm]
*A*	area in a 2-dimensional projection, [mm^2^]
*A*_r_	area ratio, [-]
*A*_ce_	ratio of area of a circumscribing ellipse, [mm^2^]
*A*_1_	area of particle, [mm^2^]
${\bar{R}}_{c3}$, ${\bar{R}}_{c7}$, ${\bar{R}}_{c28}$	average compressive strength at 3, 7, 28 days, [MPa]
*E*	modulus of elasticity, [GPa]
${\bar{E}}_{3}$, ${\bar{E}}_{7}$, ${\bar{E}}_{28}$	average Young modulus at 3, 7, 28 days, [GPa]
*R*_c_	nominal compressive strength, [MPa]
*s*	standard deviation, [-]
*c*_V_	coefficient of variation, [%]
ε	axial strain during fracture, [‰]
$\varepsilon_{T}$	transverse strain during fracture, [‰]
